# Transition from silicene monolayer to thin Si films on Ag(111): comparison between experimental data and Monte Carlo simulation

**DOI:** 10.3762/bjnano.9.7

**Published:** 2018-01-05

**Authors:** Alberto Curcella, Romain Bernard, Yves Borensztein, Silvia Pandolfi, Geoffroy Prévot

**Affiliations:** 1 Sorbonne Universités, UPMC Univ Paris 06, CNRS-UMR 7588, Institut des NanoSciences de Paris, F-75005, Paris, France

**Keywords:** Auger electron spectroscopy, scanning tunneling microscopy, silicene, silicon, silver

## Abstract

Scanning tunneling microscopy (STM), Auger electron spectroscopy (AES) and low energy electron diffraction have been used to follow the growth of Si films on Ag(111) at various temperatures. Using a simple growth model, we have simulated the distribution of film thickness as a function of coverage during evaporation, for the different temperatures. In the temperature regime where multilayer silicene has been claimed to form (470–500 K), a good agreement is found with AES intensity variations and STM measurements within a Ag surfactant mediated growth, whereas a model with multilayer silicene growth fails to reproduce the AES measurements.

## Introduction

Since their discovery in 2012 [1], silicene layers have been attracting a great interest, due to the expectation of electronic properties similar to the ones of graphene, based on theoretical studies [2]. Because of their easy synthesis, Si/Ag(111) monolayers have been intensively studied [3–6]. It has been shown that several monolayer structures can be formed, depending on the substrate temperature [7]. All of them probably correspond to a buckled honeycomb structure for Si atoms. For example, a buckling of 0.77 Å has been precisely measured for the (4 × 4) structure [8–10]. Silicene growth has also been reported on other substrates, such as Ir [11], ZrB_2_ [12]_,_ or MoS_2_ [13]_,_ although the precise crystallographic structure of these layers has not been elucidated yet.

In spite of its atomic structure close to the one of free standing silicene, silicene/Ag(111) displays different electronic properties [14–15]. This is due to a strong electronic coupling between the substrate and the silicene layer. Thus, the features in the angle resolved photoemission spectrometry (ARPES) [1], initially attributed to Dirac cones near the Fermi level, have been shown to be related to a modification of the silver band structure induced by the silicene reconstruction [14,16–19]. This strong coupling also gives rise to Si–Ag atomic exchange during the deposition of Si on the Ag(111) surface [6,20–22].

In order to avoid such strong coupling, attempts have been made to grow silicene multilayers, or "silicite" thin films, with an atomic structure similar to the one of graphite, by evaporating larger amount of Si. On Ag(111), deposition on a substrate held at 470–500 K results in the formation of successive Si layers [23–26], with an interlayer spacing of ≈3Å. Such layers display an electronic band structure, measured by ARPES, that has been interpreted as a Dirac cone located 0.25 eV below the Fermi level [27]. These layers present a metallic behavior, with an electric conductivity one order of magnitude lower than the one measured for multilayer graphene [26]. Their structure slightly differs from the one of diamond, with an interlayer spacing 1% smaller than the one found between two consecutive hexagonal buckled planes in diamond-like bulk silicon, and a Raman peak also 1% shifted from the position expected for bulk Si. They have been firstly described as a new Si allotrope, made by successive stacking of silicene layers [23–26].

However, as the surface termination presents a (√3 × √3)R30° reconstruction relative to the silicene unit cell, which is very similar to the honeycomb-chained triangle (HCT) reconstruction observed on a Ag/Si(111) surface, it has been hypothesized that the observed films could result from the growth of diamond-like Si with Ag acting as a surfactant [28]. Such conclusions were also supported by low energy electron diffraction (LEED) [29–30], ARPES [31] and grazing incidence X-ray diffraction [32]. The diamond-like structure of the film has been confirmed by scanning tunneling microscopy (STM) [33] and optical measurements [34]. The Ag termination of the surface has been also demonstrated by Auger electron spectroscopy (AES) [34], metastable atom electron spectroscopy [35] and deuterium exposure of the film [36], whereas opposite conclusions were obtained from STM observations after applying a bias pulse at low temperature [33].

Very recently, the existence of two different growth modes on Ag(111), depending on the substrate temperature, has been proposed [37–38]. At low temperature (*T* = 470 K), multilayer silicene would form, without Ag at the surface, whereas diamond-like growth would occur at high temperature (*T* = 570 K), with Ag acting as a surfactant. Thus, open questions remain on the nature of the films formed as a function of the growth temperature and on the growth mechanisms. In this paper, we have used STM, AES and LEED to follow the growth of Si films at various temperatures. Using a simple growth model, we have simulated the distribution of film thickness as a function of coverage during evaporation, for the different temperatures. In the temperature regime where multilayer silicene has been claimed to form (470–500 K), a good agreement is found with AES intensity variations and STM measurements within a Ag surfactant mediated growth, whereas a model with multilayer silicene growth fails to reproduce the AES measurements.

## Results and Discussion

**Figure 1:**
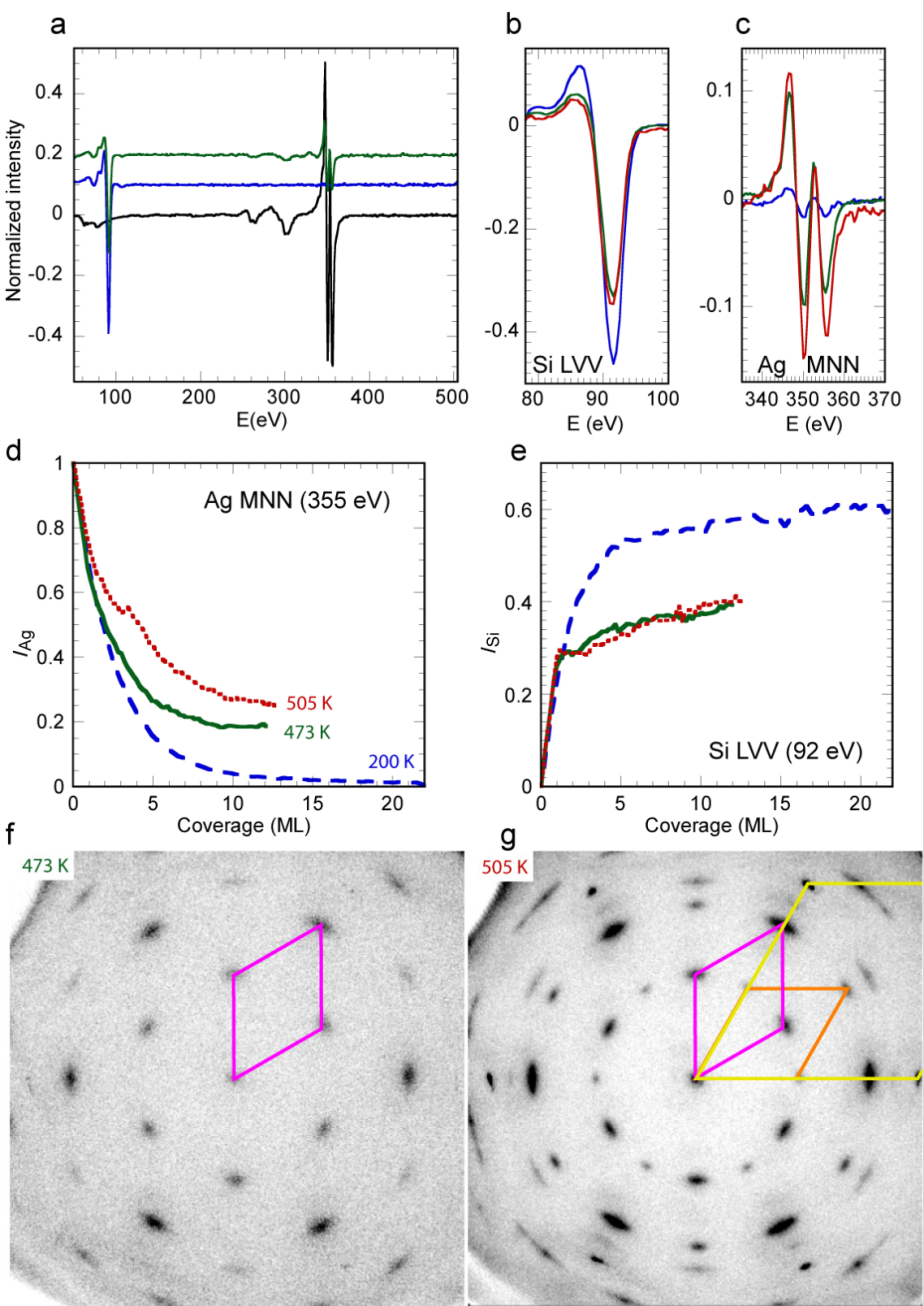
a) Full Auger spectra for the bare Ag surface (black line), after 22 ML Si evaporation at 200 K (blue line), and after 9 ML Si evaporation at 473 K (green line) - the spectra are shifted for clarity. b,c) Si LVV (b) and Ag MNN (c) signals after 12 ML Si evaporation at 200 K (blue line), 473 K (green line) and 505 K (red line). d,e) Evolution of the Ag (d) and Si (e) Auger intensities as a function of the Si coverage, for growth at 200 K (blue dashed line), 473 K (green continuous line) and 505 K (red dotted line). f,g) LEED diagrams obtained after 12 ML Si evaporation at 473 K (f) or 505 K (g), for *E* = 70 eV. The yellow lozenge is the surface unit cell of Ag(111), the purple and orange lozenges are the surface unit cells for the (√3 × √3)R30° reconstruction of Si(111).

Auger spectra taken before and after Si evaporation at the different temperatures are shown in Figure 1a–c. We have followed the peak-to-peak intensities of the Ag MNN and Si LVV transitions at 355 eV and 92 eV respectively, during growth at different substrate temperatures (*T* = 200 K, 473 K and 505 K). In the following, all intensities have been normalized to the Ag intensity measured for the clean surface prior evaporation. The evolution of the normalized Auger intensities *I*_Ag_ and *I*_Si_ is shown in Figure 1d and 1e as a function of Si coverage. For high temperature measurements, the coverage θ has been calibrated from the breaks observed in the evolution of *I*_Si_ that were attributed to the completion of a silicene monolayer. Here, one monolayer (ML) is referred to the honeycomb silicene plane, whose density is 15.7 atom·nm^−2^. This also corresponds to the atomic density of a Si(111) double plane in bulk silicon. For deposition at 200 K, the coverage has been calibrated to obtain the same value of d*I*_Si_/dθ(0) as the one measured at 473 K and 505 K.

For growth at 200 K, *I*_Ag_ decays exponentially to zero which indicates that the Si film completely covers the substrate (Figure 1d). The Si normalized intensity converges to 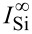 = 0.60 ± 0.04 (Figure 1e), thus corresponding to the value for a clean Si surface. Note that this value slightly differs from the one measured in [34], probably due to the different geometry used (the incidence of the electron beam with the sample normal is 30° here instead of 45° in [34]). After growth at 200 K, the LEED diagram showed only a diffuse background, which indicates that the film is amorphous. On the contrary, for growth at higher temperature, *I*_Ag_ does not decay to zero (Figure 1d), but to a value 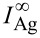 = 0.185 ± 0.02 for *T* = 473 K and 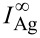 = 0.25 ± 0.04 for *T* = 505 K. Moreover, *I*_Si_ saturates at a value lower than 0.6, namely 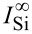 = 0.40 ± 0.04 for both temperatures. These two observations demonstrate that the surface is not a thick continuous pure Si layer.

Figure 1f and Figure 1g show the LEED diagrams measured at room temperature after 12 ML deposition at 473 K and 505 K. They both display the spots of the Si (√3 × √3)R30° reconstruction, associated with a single epitaxial relationship for *T* = 473 K, corresponding to 
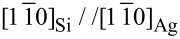
 and with a minority epitaxial relationship for *T* = 505 K, corresponding to 
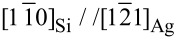
.

For growth at *T* = 473 K, the substrate spots are practically no more visible, and the LEED intensity from these spots is around three order of magnitude less than the value measured on the bare surface. Thus, the measured Ag Auger intensity, which is equal to 20% of its initial value, cannot be due to part of the surface covered by very thin Si layers. Note that these results are completely at variance from those obtained by LEED and AES by De Padova et al. [37]: after evaporation of 10 ML Si at 473 K, they have observed that the ratio of the Si and Ag Auger intensities was very small, namely *I*_Ag_/*I*_Si_ = 0.09, instead of *I*_Ag_/*I*_Si_ = 1.16 for the silicene monolayer. On the contrary, the most intense spots on their LEED diagram were the substrate spots. From that, they concluded to an imperfect wetting of the 10 ML film.

Coming back to the present results, our LEED diagram obtained after growth at *T* = 505 K shows results quite different from the 473 K ones. Substrate spots are clearly seen after deposition of 12 ML. The high intensity of the substrate spots shows that the film does not cover the whole surface homogeneously. As a consequence, the substrate must also significantly contribute to the AES signal measured at the end of growth, which is indeed larger than its value for 473 K (Figure 1d).

**Figure 2:**
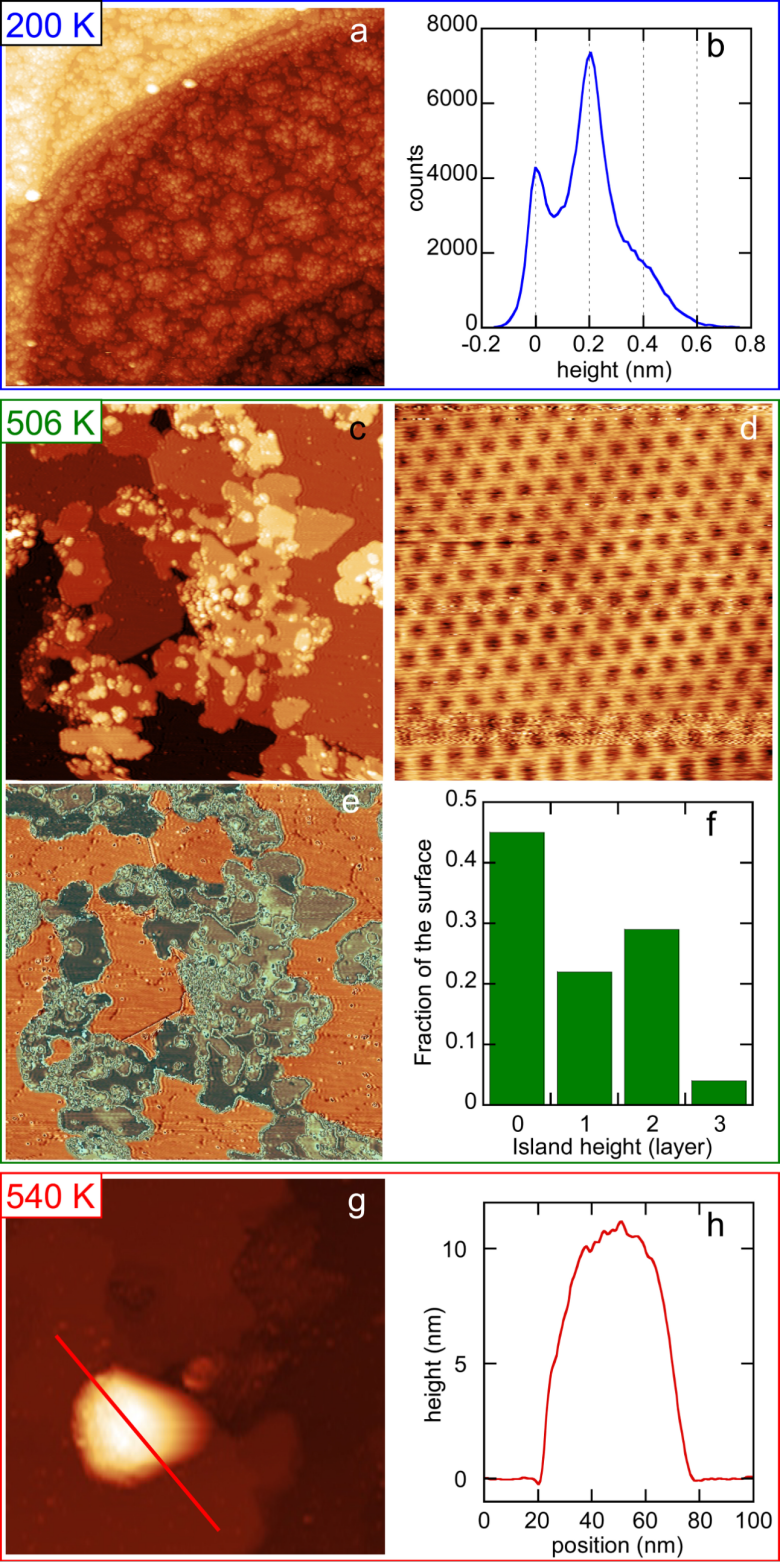
STM images of the Ag(111) surface after evaporation of 1 ML Si at 200 K (a), 2.2 ML Si at 506 K (c,e), ≈3 ML Si at 540 K (g). e) shows the apparent height of the surface modulo the Ag step height. Size of the images 170 × 170 nm^2^. b) and f) Distribution of heights extracted from the large terrace in image (a) and from image (e) respectively. d) Detailed view (8.4 × 8.4 nm^2^) of the Si film with (√3 × √3)R30° reconstruction, for growth at 506 K. h) Height profile along the line shown in (g).

In Figure 2 are presented STM images of the surface after Si growth at different temperatures. Figure 2a shows the surface after evaporation of 1 ML Si at 200 K. In Figure 2b is shown the corresponding distribution of apparent height. Even if, for this low deposition temperature, the film is amorphous, the distribution shows a clear peak at 0.20 nm characteristic of the apparent height of a first Si layer, and a shoulder at 0.40 nm characteristic of a second Si level. The peak at zero corresponds to the Ag surface. For this evaporated quantity, the surface is not fully covered with Si, since nearly one quarter of the surface remains free. This indicates that the second layer starts to grow before completion of the first one, corresponding to a rough growth mode of the amorphous film.

For growth at 506 K, a similar behaviour is observed, but after completion of the monolayer: above 1 ML, thicker islands grow, with a (√3 × √3)R30° reconstruction, in a rough growth mode, as shown in Figure 2c for 2.2 ML. In Figure 2e is presented, for the same area, the apparent height of the surface modulo the Ag step height *h*_Ag_. For clarity, if one applies this algorithm to the bare Ag surface, all terraces appear at the same *z* level. For the Si/Ag film, islands with same thickness appear at the same *z* value in the [0, *h*_Ag_[ interval. This allows to display, with a same color, Si islands of same thickness, independently of the initial Ag terrace where they have grown. In Figure 2e, brown regions correspond to Si islands. The large flat orange domains correspond to the silicene monolayer. For such coverage (2.2 ML), the monolayer covers 45% of the surface. In Figure 2g is shown the surface after evaporation of ≈3 ML at 540 K. In that case, in addition to large flat islands, very thick islands also form. For example, the apparent height of the island shown in Figure 2g is 11 nm. Note that the silicene monolayer has not dewetted for this growth temperature and that a large part of the surface is covered by this layer.

In order to discriminate between the different growth models, and to determine if the differences observed between Si growth in the 470–540 K temperature range result from two different growth modes, we have computed the evolution of the distribution of film thickness during evaporation, in the frame of a Monte Carlo simulation, and compared the results to the AES data. In the model chosen, the film is constituted by different terraces of various heights *h*_i_ = *i* × *d*_0_ where *d*_0_ is the Si interlayer spacing. For the *N*_pt_ experimental points corresponding to the various coverages, the distribution of the terrace heights is used to calculate the Auger intensity. This is done by assuming either a surface termination for the silicon film similar to the Ag-induced (√3 × √3)R30° Si(111) reconstruction, or a pure Si termination. The Auger intensities have been computed using effective attenuation lengths [39] (EAL) for electrons, that have been fitted to obtain the best agreement with the experiments, and are obviously kept fixed for all experiments. The normalized Auger intensities for Ag and Si are given by the following equations in the framework of no Ag surfactant layer:


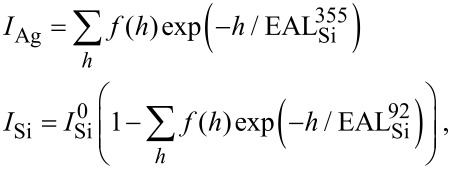


whereas in the framework of a surfactant Ag layer, they are given by:


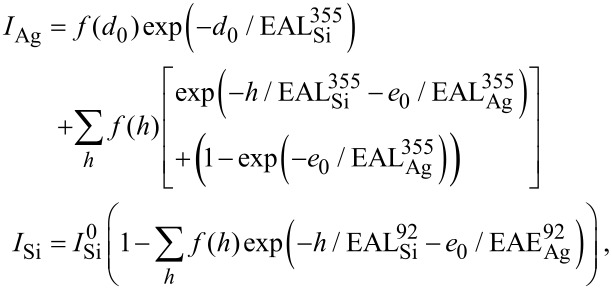


Where *e*_0_ = 0.13 nm is the equivalent thickness of the surfactant Ag layer, *f*(*h*) is the fraction of the surface covered by a Si layer of thickness *h*, and 
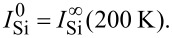
 The values of the EAL for 355 eV and 92 eV electrons through a Si layer, 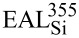 and 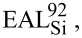 have been set to obtain the best agreement with Auger data in the linear submonolayer regime. In the model of surfactant Ag, the attenuation lengths through Ag layers have been set to obtain the best agreement for the values of 
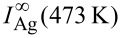
 and 
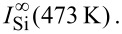
 Note that they do not play any role in the case of Ag-free growth. All EAL values are given in Table 1. They are in a relative good agreement with computed values of inelastic mean free path (IMFP) [40], taking into account the fact that the attenuation length should be less than the IMFP due to a collection angle less than 90° for the escaping electrons, and the large uncertainty related to the computation of the IMFP [40].

**Table 1:** Effective attenuation lengths fitted from the Auger signals, and comparison with calculated IMFP [40].

	fitted values (nm)	IMFP (nm)

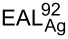	0.32	0.51
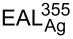	0.72	0.71
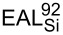	0.52	0.48
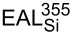	0.76	1.14

The comparison between simulated (*I*_th_) and experimental (*I*_exp_) intensities provides the value of


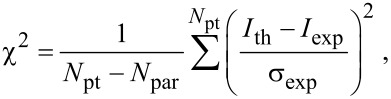


associated with the model of the simulation, *N*_par_ being the number of free parameters. This process is used to adjust the parameters of the model until a minimum of χ^2^ is reached.

At each step of the simulation, an evaporated Si atom arrives on a terrace of height *h_i_* with a probability proportional to the fraction of the surface covered by the terraces of such height *f*(*i*), giving rise to a variation δθ of the coverage. This atom has then the probabilities *p_n_*(*i*), *p_d_*(*i*), *p_u_*(*i*) to either nucleate on this terrace, diffuse to a lower terrace or to an upper terrace. For simplicity, we assume that these probabilities are proportional to the fraction of the surface covered with terraces of height *h_i_*, *h_i_*_−1_ and *h_i_*_+1_ respectively, with proportion factors equal to α*_n_*(*i*), α*_d_*(*i*), α*_u_*(*i*). They are thus given by:


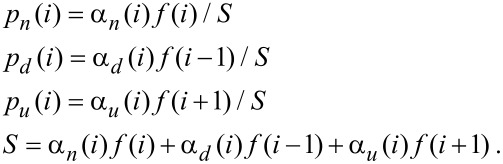


The atom stops diffusing when it nucleates. In the frame of multilayer silicene growth, above the monolayer, nucleation gives rise to the growth of successive terraces. In the frame of silicon growth mediated by surfactant Ag atoms, the nucleation above the Si monolayer is treated differently, as it gives rise to the formation of thick Si islands with an additional layer corresponding to the Ag–Si HCT reconstruction on top of the island. In that case, nucleation of an atom on the monolayer results in the growth of an island of height *h*_min_, covering a fraction δθ of the surface, by conversion of a fraction δθ(*h*_min_ + 0.5) of the monolayer into a thick island, and *p_u_*(0) becomes the probability for an atom on the substrate to jump on. Note that we have taken into account the fact that the HCT reconstruction corresponds to an additional Si amount of 0.5 ML. Note also that multilayer silicene growth would correspond to *h*_min_ = 2, without HCT layer. With no loss of generality, we have set α*_n_*(*i*) = 1. The free parameters of the model are thus the values of α*_d_*(*i*), α*_u_*(*i*), *h*_min_. For each temperature, α*_d_*(*i*) and α*_u_*(*i*) have been chosen constant, except for the diffusion between the substrate and the silicene layer, and between the silicene layer and the thicker islands in the framework of Ag-surfactant growth.

All kind of growth modes can be simulated using these parameters. For example, setting α*_u_*(*i*) = 0 and α*_d_*(*i*) >> 1 leads to a classical 2D or Frank–van der Merwe growth mode. Setting α*_d_*(*i*) = 0 and α*_u_*(*i*) >> 1 leads to a classical 3D or Volmer–Weber growth. Stranski–Krastanov growth is obtained with α*_u_*(*i* < *i_c_*) = 0, α*_d_*(*i* ≤ *i_c_*) >> 1, α*_u_*(*i* ≥ *i_c_*) >> 1 and α*_d_*(*i* > *i_c_*) = 0, where *i_c_* is the critical thickness above which 3D islands form.

**Figure 3:**
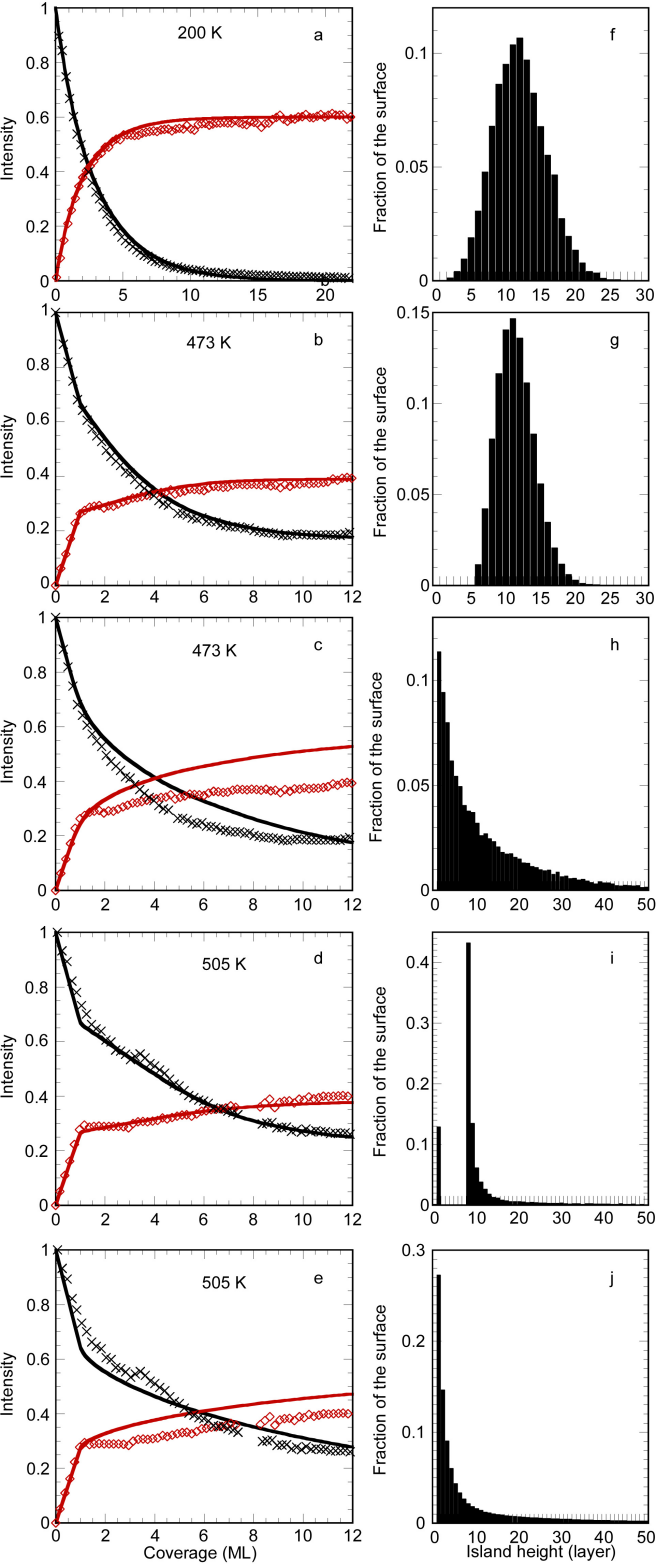
a–e: Evolution of the Ag (black crosses and black line) and Si (red lozenges and red lines) Auger intensities as a function of the Si coverage. Comparison between experiments (symbols) and best fits (lines) for growth at 200 K (a), 473 K (b,c) and 505 K (d,e). Fits for a), c) and e) are performed without surfactant Ag atoms, whereas fits for b) and d) are performed in the framework of a surfactant Ag layer. In f–j are drawn the corresponding histograms of the island heights at the end of the simulation.

Figure 3 presents the comparison of the experimental and simulated AES intensities for the three temperatures studied. The corresponding parameters of the simulation are given in Table 2.

**Table 2:** Parameters of the Monte Carlo simulation corresponding to the fit of the AES data shown in Figure 3a–e.

	200 K (a)	473 K (b)	473 K (c)	505 K (d)	505 K (e)

surfactant Ag	No	Yes	No	Yes	No
α*_u_* (0)	0	0	0	0	0
α*_u_* (1)	0.2	0.13	500	1	20
α*_u_*(*i* > 1)		0	10	12	23
α*_d_*(1)	1	1000	1000	1000	1000
α*_d_*(*h*_min_)	0.05	0.23	1	5	0
α*_d_*(*i* > *h*_min_)		0.23		0.5	
*h*_min_	2	2	2	8	2

For *T* = 200 K, (Figure 3a) a good agreement is found with a model of random growth, with no surfactant Ag, with a medium probability for an incoming atom to diffuse from the first Si layer to the substrate (α*_d_*(*i*) = 1), the other values of α*_d_*(*i*) and α*_u_*(*i*) being small. The distribution of island height after 12 ML Si evaporation at 200 K is shown in Figure 3f and is very close to the one derived from the binomial distribution, i.e., if the α*_d_* and α*_u_* coefficients are set to zero, with variance equal to 14.4 instead of 12. For such temperature, the notion of layer for the amorphous film is no more adequate, excepted for the very first layers. We have checked that the level of discretization used in the simulation did not change the final results.

A good agreement is also obtained between AES experimental and simulated intensity evolutions for *T* = 473 K and *T* = 505 K, if one assumes that Ag acts as a surfactant for the growth of Si/Ag(111) (Figure 3b and Figure 3d). The fits have been obtained with different probabilities for the growth parameters, which obviously depend on the temperature. Note that the α*_d_*(1) coefficient is set to an arbitrary high value to ensure the continuous wetting of the substrate by the silicene layer.

For *T* = 473 K, a good fit is obtained if one now assumes a conversion of monolayer to Si islands having a height of 2 layers and covered with Si and Ag atoms forming the (√3 × √3)R30° reconstruction. There is a small probability to diffuse towards the lower terraces (α*_d_*(*i* > 2) = 0.23), whereas the probability to diffuse towards the upper terraces is zero. This results in a narrow distribution of film thickness, as shown in Figure 3g, where the variance of the distribution is equal to 7.7. The simulation also predicts that, at the end of the growth, the surface is fully covered by Si islands of thickness larger than 5 layers. This explains why the intensity of the LEED (1 × 1) spots of the substrate is very weak (Figure 1f). For this temperature, the simulation corresponds thus to an imperfect layer by layer growth mode [41] for which, after the completion of the silicene layer, the *n* + 1 layer starts to grow before completion of the *n* layer.

For *T* = 505 K, the best fit is obtained by conversion of the monolayer to similar islands having a height of 8 ML. At this temperature, there is a high probability, for the atoms in the islands, to diffuse towards the higher terraces (α*_u_*(*i* > 1) = 12), which results in the growth of thick islands, with a very wide distribution of film thickness (see Figure 3i). The simulation also predicts that, at the end of the growth, 13% of the surface remains covered by the silicene monolayer, which explains why the (1 × 1) spots of the substrate remains visible in Figure 1g.

On the contrary, no good fit can be obtained in the frame of a multilayer silicene growth. The best fits obtained in such framework are shown in Figure 3c for *T* = 473 K and in Figure 3e for *T* = 505 K. As it is clear from the poor agreement, this model fails to reproduce the behaviour observed for the Auger intensities above 1 ML. For both temperatures, the increase of the Si intensity is too small. For *T* = 473 K, the predicted decay of the Ag intensity is too slow whereas for *T* = 505 K, the complex behaviour of the signal cannot be reproduced. Both fits correspond to the formation of thick 3D islands above the monolayer as can be observed from the distributions shown in Figure 3h and Figure 3j.

**Figure 4:**
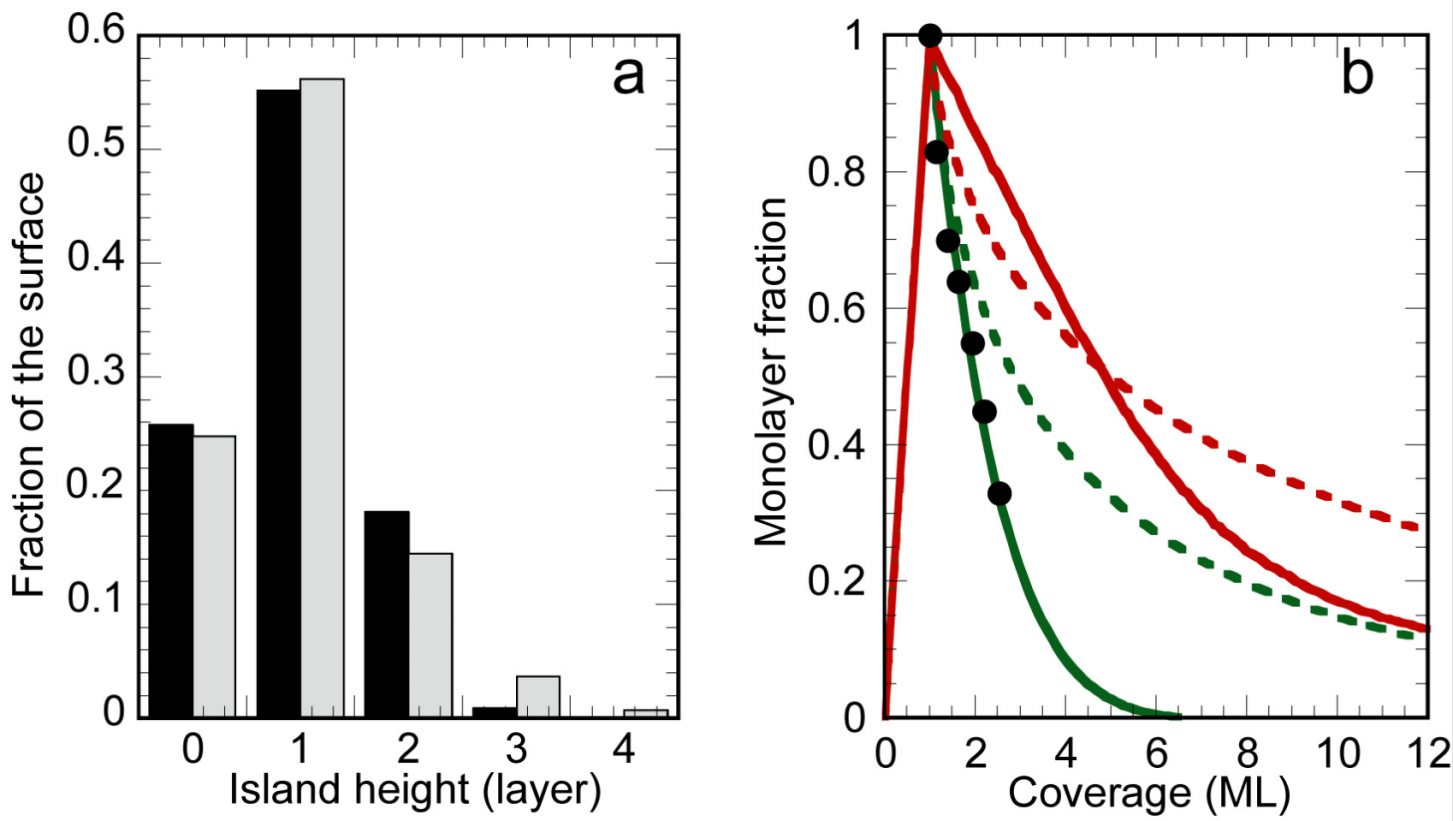
a) Histogram of the island heights for growth of 1 ML at 200 K. Comparison between the results of the growth simulation (grey) and the experimental values measured on the STM image shown in Figure 2a (black). b) Evolution of the surface fraction covered by the monolayer. Black dots: STM measurements during growth at 506 K. Green and red lines: results of growth simulations fitted on AES data at *T* = 473 K and *T* = 505 K respectively, in the framework of surfactant Ag (continuous lines) or not (dotted lines).

It is also interesting to compare the simulation results with the LEED and STM observations. Figure 4a presents the comparison of the distribution of film thickness for 1 ML deposition at 200 K obtained by the Monte Carlo fit in comparison with the STM data obtained from the analysis of the STM image shown in Figure 2a. A very good agreement is found between the experimental and simulated distributions.

In Figure 4b is drawn the evolution of the surface area covered by the silicene layer as a function of Si coverage, for growth at 473 K and 505 K. For both temperatures, in a model of surfactant Ag (continuous lines), it first increases linearly up to 1 ML, and then decays with a decay length of 1.6 ML for *T* = 473 K and 7.8 ML for *T* = 505 K. A very good agreement is obtained between STM results for growth at 506 K, and growth simulations fitted on AES data at 473 K, in the framework of surfacting Ag atoms. On the contrary the simulations performed for AES data at 505 K indicate that a larger fraction of the surface remains covered by the monolayer. The simulations also predict the formation of thick islands after the completion of the monolayer, which are not observed by STM at such temperature, but are observed for growth at 540 K. For example, the height of the island shown in Figure 1g corresponds to around 35 Si layers. No correct agreement is found with the results of AES simulations performed in the framework of multilayer silicene growth (without surfactant Ag, dotted lines).

Thus, both STM and AES experiments indicate that as the temperature increases, a transition is observed between an imperfect layer-by-layer growth and a Stranski–Krastanov growth mode (*i_c_* = 1). This transition is observed by AES between 473 K and 505 K, and between 506 K and 540 K by STM. These differences may be due to the experimental uncertainties on the temperature measurements or to the different evaporation rates used during the experiments.

## Conclusion

The quantitative analysis of the evolution of AES intensity during Si growth at different temperatures shows that the growth mechanism is different for low temperature deposition (*T* = 200 K) and in the regime described previously as "intermediate" (473–505 K). In particular, low temperature deposition results in a rough growth mode, with no mobility of Ag atoms. A very good agreement is obtained between AES and STM measurements for the distribution of island heights in a model with little interlayer diffusion. On the contrary, growth at intermediate temperatures results in a Ag surfactant mediated Si growth. A good agreement with AES, LEED, and STM measurements is obtained by considering that the Si islands film is terminated by the Ag/Si(111) (√3 × √3)R30° reconstruction. As temperature increases, thicker Si islands form and the film becomes more and more inhomogeneous, resulting in a larger fraction of the surface uncovered by the thick Si islands.

## Experimental

Experiments were performed in two UHV set-ups with 10^−10^ mbar base pressure. The Ag(111) sample was cleaned by series of cycles of Ar ion sputtering at 0.6 keV followed by annealing at 850 K. Si was evaporated from a Si rod with a commercial Omicron EFM3 evaporator. For AES/LEED experiments, the evaporation rate was between 0.03 and 0.06 ML/min, whereas for STM experiments, it was around 0.004 ML/min. Auger peak-to-peak intensities were measured during growth with a Riber CMA Auger spectrometer working at 3 keV primary beam, 30° incidence, using a lock-in amplifier at 1 kHz with 0.4 V modulation amplitude. LEED patterns were obtained with an Omicron SPA-LEED apparatus, at ambient temperature after evaporation. STM images were obtained during growth with an Omicron VT-XA STM. Image corrections were performed using a home-made software described elsewhere [42]. Temperature measurements were performed with a thermocouple located on the sample heating stages, previously calibrated with another thermocouple soldered on the surface of a testing sample. As a result, if for a given sample in either set-up the precision on the reproducibility of the measure is of the order of 1K, the uncertainty on the absolute value of the temperature may be of the order of 10 to 20 K at 500 K. The previous analysis shows indeed that the measured temperature in the STM set-up is likely overestimated with respect to the one in the AES/LEED set-up.
